# Annotations

**Published:** 1896-07-04

**Authors:** 


### Annotations.

### The Ramifications of "Notification."

Attention has already been called to the anomalous provisions contained in the Factory and Workshops Act, 1895, according to which medical practitioners have to notify cases of lead, phosphorus, or arsenical poisoning, or anthrax, to the Inspector of Factories, while all other notifications go to the sanitary authorities. This is a matter which will require very careful watching in the future. It is but the thin edge of a large wedge, and already we find in the report of H.M. Chief Inspector of Factories the following suggestion made by one of the inspectors: "This very excellent provision of the new Act might, I think, be extended so as to make it compulsory for all house surgeons of hospitals, etc., and even general medical practitioners, to send notice to H.M. Chief Inspector of all cases of accident occurring in a factory or workshop treated by them. We should in this way obtain a more complete return of accidents than under the existing method." Of course! Nothing easier than to put all these matters on the medical man. It should be definitely understood, however, that notification in all such cases stands on an entirely different footing from the notification of infectious diseases. The latter is done for the public good—for the control of diseases which tend to spread indifferently through the whole mass of the population. For the sake of this great public good, medical men are compelled, along with the householder in whose house the case occurs, who is equally liable by law, to give information to an authority which is appointed to look after the health of all alike. These notifications under the Factory Act are quite a different affair; they do not touch the greater public at all, and are a mere means of facilitating the supervision of the inspectors over certain classes of factories. Any extension of the system of notification in this direction should be firmly opposed. The next thing will be that surgeons will be asked to report to the police every broken head they dress, merely for the sake of facilitating the supervision of the public-houses and drinking shops!

### Ignorant Editors and Struggling Hospitals.

Readers of newspapers must sometimes wonder whether editors exercise any control over the shoals of complaining letters which appear in their columns. From the absurdity of many of these complaints we might think that they are shovelled in as they come. As a specimen of the worthless and ignorant letters which are allowed to appear in certain papers, we might allude to one in the *Echo* of June 20th, in which "A Suffering Working-man" complains that having applied to four separate hospitals he has failed to obtain admission at any of them. He writes as follows:—"Applicants are being continually told 'we have no beds,' but what, I ask, is being done with the enormous sums which are being continually given to the hospitals? I am told, and on good authority, that the staff of these institutions live in ease and luxury on the money which is given by the generous public, whilst the welfare of the patients is a secondary consideration. I think therefore, sir, it is high time that this matter was thoroughly looked into." Cannot the editor of a paper which plays the rôle of the working man's friend and the supporter of all that is true and righteous see how scandalously untrue is the suggestion made in this letter, and how injurious it must be to working men to divert funds from the hospitals, as such aspersions, lies though they be, always tend to do? Surely an editor should take the trouble to let some one or other on his staff find out something about the management of hospitals, and save his paper the ridicule which must attach to such comments upon hospital matters?

### The Plague Spots of London.

The interesting spot maps which year after year are issued by the Metropolitan Asylums Board, showing the residence of each case of each of the notifiable infectious disorders, point with unerring finger to certain localities as the natural homes of these diseases. It cannot be by accident that particular districts stand out black in map after map, blackened by the close aggregation of the dots, each of which indicates the locality in which a case of infectious disease originated. What sanitary inspectors, and district medical officers, and parochial clergy, know by daily experience, these maps demonstrate as the result of carefully compiled statistics, that certain areas are peculiarly prone to the development of zymotic disease, and are in a manner the plague spots of London. Interesting, however, as these maps are in pointing out the natural and almost constant homes of infection, they by no means tell us where infectious maladies take on their most virulent form. It might by some be taken for granted that where a disease prevails the most there the most would die from it, and that is often true; but the proportion who die, out of those attacked, varies enormously. A writer in the *British Medical Journal* for June 20th points out that, taking London by parishes, the mortality of scarlet fever in different districts ranges from 1·7 per cent. to 11·7 per cent. of the cases attacked; and that in regard to diphtheria the case mortality goes in some districts as low as 9 per cent., and in others as high as 45 per cent. of those affected by the disease. What is the cause of this extreme variation? The figures quoted above are taken from the returns of whole parishes, areas which are far too large for purposes of comparison. But the spot maps of the Asylums Board show that the areas of greatest prevalence are comparatively small patches, and the probabilities are that the areas of high fatality are equally small patches, and that in them a far higher case mortality prevails than is indicated even by the figures mentioned above. We need hardly say that a careful statistical inquiry into this important question would well repay the trouble expended on it. It would point out the real plague-spots of London, for everything tends to show that virulence of type is even more important than excessive prevalence. The occurrence of infectious disease is often a matter beyond the control of sanitary officials; quite otherwise, however, is it with those insanitary conditions by which comparatively simple ailments are changed into mortal diseases. For these they are responsible.

